# Diabetes, pancreatic cancer, and metformin therapy

**DOI:** 10.3389/fphys.2014.00426

**Published:** 2014-11-07

**Authors:** Jun Gong, Lori A. Robbins, Aurelia Lugea, Richard T. Waldron, Christie Y. Jeon, Stephen J. Pandol

**Affiliations:** ^1^Department of Medicine, Cedars-Sinai Medical CenterLos Angeles, CA, USA; ^2^Department of Medicine, Veterans AffairsLos Angeles, CA, USA; ^3^Cancer Prevention and Genetics, Samuel Oschin Comprehensive Cancer Institute, Cedars-Sinai Medical CenterLos Angeles, CA, USA; ^4^Department of Medicine, David Geffen School of Medicine, University of California-Los AngelesLos Angeles, CA, USA

**Keywords:** metformin, pancreatic cancer, diabetes, mTOR, AMPK, insulin, IGF-1

## Abstract

Pancreatic cancer carries a poor prognosis as most patients present with advanced disease and preferred chemotherapy regimens offer only modest effects on survival. Risk factors include smoking, obesity, heavy alcohol, and chronic pancreatitis. Pancreatic cancer has a complex relationship with diabetes, as diabetes can be both a risk factor for pancreatic cancer and a result of pancreatic cancer. Insulin, insulin-like growth factor-1 (IGF-1), and certain hormones play an important role in promoting neoplasia in diabetics. Metformin appears to reduce risk for pancreatic cancer and improve survival in diabetics with pancreatic cancer primarily by decreasing insulin/IGF signaling, disrupting mitochondrial respiration, and inhibiting the mammalian target of rapamycin (mTOR) pathway. Other potential anti-tumorigenic effects of metformin include the ability to downregulate specificity protein transcription factors and associated genes, alter microRNAs, decrease cancer stem cell proliferation, and reduce DNA damage and inflammation. Here, we review the most recent knowledge on risk factors and treatment of pancreatic cancer and the relationship between diabetes, pancreatic cancer, and metformin as a potential therapy.

## Introduction

### Epidemiology

Pancreatic cancer is the twelfth most common cancer in the US but represents the fourth leading cause of cancer death in both men and women (Howlader et al., 2014). The prognosis is extremely poor with a 5-year survival rate of only 6.7% as pancreatic cancer is usually asymptomatic in the early stages of disease and most cases are diagnosed relatively late (Howlader et al., 2014). Treatment and advances in early detection are of crucial importance.

### Risk factors

Smoking is a well-known risk factor for pancreatic cancer and is estimated to contribute to 20–30% of all cases of pancreatic cancer (Iodice et al., 2008). A meta-analysis including 82 studies showed that smokers have a 75% increased risk of pancreatic cancer compared to non-smokers and that the increased risk persists at least 10 years after smoking cessation (Iodice et al., 2008). A meta-analysis from 2012 suggested that risk of pancreatic cancer initially increases with cigarette amount but levels off at higher intensities of cigarette smoking, indicating that quantity has some role in determining risk (Zou et al., 2014).

A meta-analysis from 2010 found that individuals with chronic pancreatitis had a 13.3-fold higher risk of developing pancreatic cancer and a 5.8-fold increased risk after excluding cases diagnosed within 2 years of cancer diagnosis (Raimondi et al., 2010). However, despite this strong relationship, only about 5% of patients with chronic pancreatitis will actually develop pancreatic cancer in a 20 year period (Raimondi et al., 2010). Hereditary pancreatitis is a rare autosomal dominant disease due to a mutation in the gene encoding trypsinogen in which patients develop chronic pancreatitis at a young age (under 30). The cumulative risk of developing pancreatic cancer is 40% by age 70 (Lowenfels et al., 1997).

A recent meta-analysis which evaluated risk based on different intensities of alcohol consumption provided evidence that heavy alcohol consumption (defined as >3 drinks per day) increases risk for pancreatic cancer by 22%, independent of tobacco use, whereas moderate alcohol consumption did not carry an increased risk (Tramacere et al., 2010).

Another important risk factor is body mass index, which has been associated with an elevated risk of pancreatic cancer in several studies (Larsson et al., 2007; Arslan et al., 2010; Jiao et al., 2010; Genkinger et al., 2011). In a pooled analysis of 14 cohort studies, risk for pancreatic cancer was 47% greater for individuals with BMI>30. Higher waist to hip ratio was also found to be positively associated with pancreatic cancer, suggesting that central obesity in particular may confer risk (Genkinger et al., 2011). Lastly, a recent meta-analysis involving more than 3-million individuals identified tobacco use, obesity, and heavy alcohol, among a host of other factors, as the 3 most important risk factors for pancreatic cancer while vegetable and fruit consumption offered the greatest protection against pancreatic diseases (Alsamarrai et al., 2014).

## Diabetes and pancreatic cancer

### Diabetes mellitus as a risk factor for pancreatic cancer

Diabetes mellitus (DM) or glucose intolerance may be present in up to 75% of patients with pancreatic cancer, a figure much higher than in other cancer types in whom the prevalence is no more than 30% (Permert et al., 1993b; Aggarwal et al., 2013). The relationship between DM and pancreatic cancer is bi-directional, as studies point to both increased risk of pancreatic cancer in those with long-term diabetes, as well as greater incidence of diabetes in sync with the development of pancreatic cancer (Li, 2012). Many studies evaluating DM as a risk factor have focused on patients with DM diagnosed several years prior to the time of pancreatic cancer diagnosis in order to exclude cases of DM that are a result of pancreatic cancer. This follows from the assumption that pancreatic cancer is rapidly fatal and therefore DM diagnosed several years prior to cancer diagnosis would unlikely be from the cancer (Li, 2012).

In a recent pooled analysis, after adjusting for age, gender, prior involved study, alcohol use, smoking, BMI, and family history of pancreatic cancer, patients with DM had a 40% increased risk of pancreatic cancer (Elena et al., 2013). This analysis excluded cases developing within 2 years, providing evidence for DM as a risk factor rather than just a result of pancreatic cancer. A meta-analysis of 20 studies conducted in 1995 showed that patients with DM for 5 or more years had a two-fold increased risk (Everhart and Wright, 1995). In another 2005 meta-analysis which included 36 studies, individuals with DM for >5 years had a 50% increased risk of pancreatic cancer (Huxley et al., 2005). Diabetes is characterized by hyperglycemia and insulin resistance, which can both contribute to tumor formation. In a prospective nested case-control study, higher levels of proinsulin, a marker of peripheral insulin resistance, was found to be associated with pancreatic cancer, independent of hemoglobin A1c, suggesting that insulin resistance may be a stronger carcinogenic contributor than hyperglycemia (Wolpin et al., 2013). This finding was supported by the fact that lower levels of adiponectin, which functions to enhance insulin sensitivity, was associated with increased pancreatic cancer risk (Bao et al., 2013).

### Mechanisms of risk

**Figure 1:**
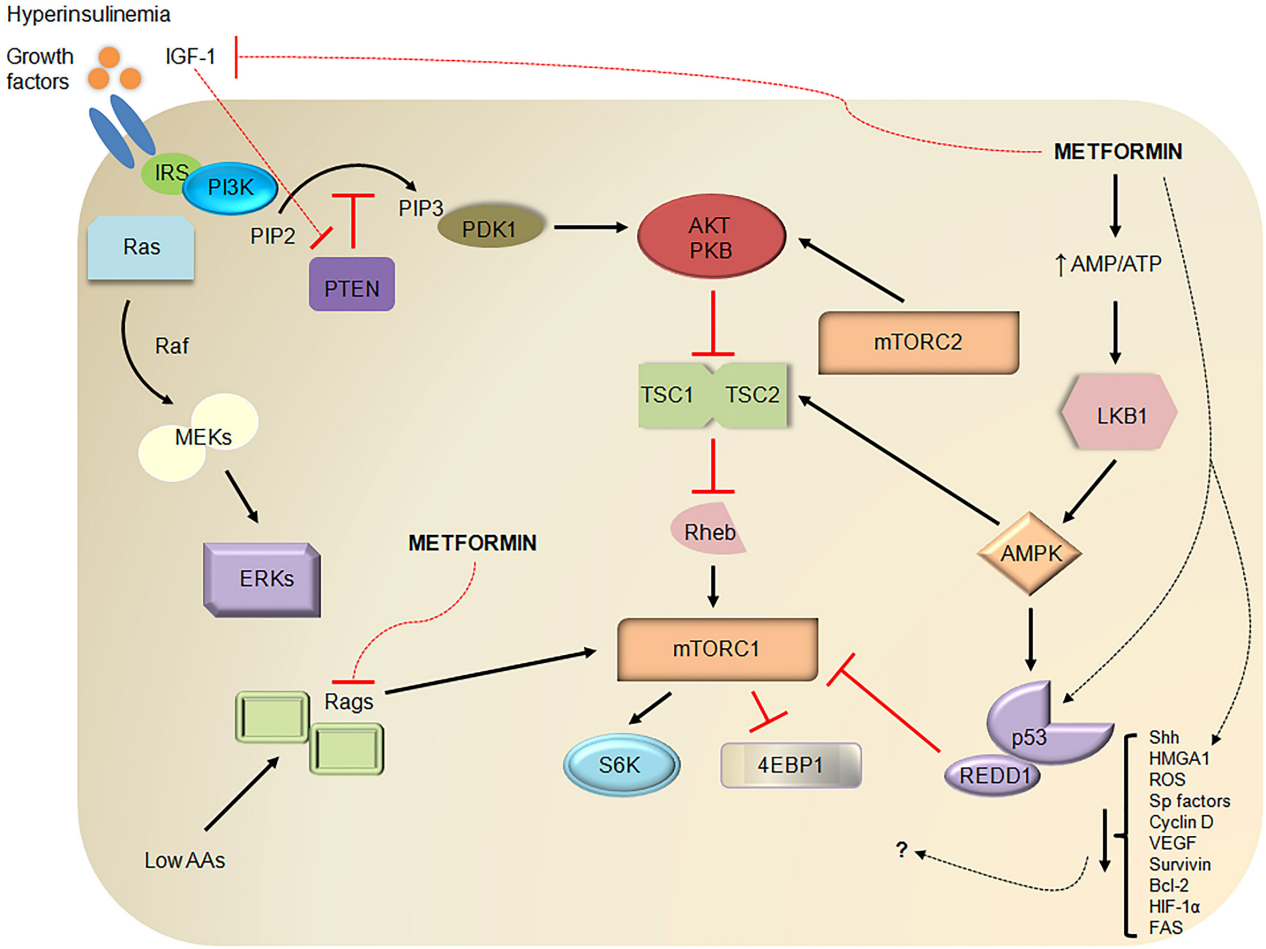
**Metformin demonstrates antitumor properties through several pathways**. Diabetes mellitus type 2 (DM2) is often characterized by insulin resistance, hyperglycemia, and compensatory hyperinsulinemia. Insulin increases IGF-1 levels by displacing IGF-1 from common binding proteins, stimulating hepatic growth hormone signaling, and decreasing IGF-binding protein production. Like other growth factors, insulin and IGF-1, upon binding to their respective growth factor receptors, can promote pancreatic cancer development through MAPK/ERK or Ras/Raf/MEK/ERK signaling and PI3K/Akt/mTOR signaling. For example, IGF-1 binding to the IGF-1 receptor recruits and activates PI3K via adaptor proteins such as IRS, converts PIP2 to PIP3 (a process that is inhibited by PTEN), activates Akt/PKB through PDK1- and mTORC2-mediated phosphorylation, and inhibits formation of TSC1-TSC2 thereby releasing its inhibition on Rheb (an mTORC1 activator). Activated mTORC1 is a key regulator of cell growth, metabolism, survival, and proliferation through downstream mediators such as 4EBP1 and S6K. Metformin has been known to uncouple the electron transport chain at complex I leading to decreased ATP production and activation of LKB1 and AMPK. AMPK is a stabilizer of TSC1-TSC2 and activator of p53, a tumor suppressor. Independent of AMPK, metformin increases p53-dependent expression of REDD1, an inhibitor of mTORC1, and inhibits mTORC1 by inhibiting Rags. Metformin also reduces hyperinsulinemia and IGF-1 levels and offers further antitumor effects by reducing levels of Shh, HMGA1, ROS, Sp transcription factors, Sp-related oncogenic proteins (cyclin D1, VEGF, survivin, Bcl-2, FAS), and HIF-1α through relatively unknown mechanisms. Dashed lines represent putative or suggested pathways while red lines represent inhibitory pathways. IGF-1, insulin-like growth factor-1; IRS, insulin receptor substrate; PI3K, phosphoinositide 3-kinase; PIP2, phosphatidylinositol-4,5-bisphosphate; PTEN, phosphatase and tensin homolog; MAPK, mitogen-activated protein kinase; MEKs, MAPK kinases; ERKs, extracellular signal regulated kinases; PDK1, phosphoinositide-dependent kinase 1; PKB, protein kinase B or Akt; TSC1, tuberous sclerosis complex 1; Rheb, Ras homolog enriched in brain; mTOR, mammalian target of rapamycin; 4EBP1, eukaryotic initiation factor 4E binding protein 1; S6K, S6 kinase; AMP, adenosine monophosphate; LKB1, liver kinase B1; AMPK, AMP-activated protein kinase; AAs, amino acids; Rags, Rag GTPases; REDD1, regulated in development and DNA damage responses 1; Shh, Sonic hedgehog; HMGA1, high mobility group AT-hook 1; ROS, reactive oxygen species; Sp, specificity protein; VEGF, vascular endothelial growth factor; Bcl-2, B-cell lymphoma-2; HIF-1α, hypoxia-inducible factor-1 alpha; FAS, fatty acid synthase.

DM and associated obesity may lead to increased risk for cancer through several mechanisms (Figure 1). Individuals with DM2 often have peripheral insulin resistance and develop compensatory hyperinsulinemia (Godsland, 2009). Insulin is a growth promoting hormone and acts by increasing cell proliferation, decreasing apoptosis, increasing glucose utilization, and enhancing responsiveness to other growth factors; all of these actions are important for cancer progression (Ding et al., 2000; Draznin, 2011). Insulin also decreases insulin-like growth factor (IGF) binding protein production thereby increasing the amount of bioavailable IGF-1 (Powell et al., 1991). IGF-1 is a more potent mitogen than insulin and promotes pancreatic cancer cell proliferation and invasion while inhibiting the tumor suppressor phosphatase and tensin homolog (PTEN, Ma et al., 2010). IGF-1 receptor binding leads to activation of the PI3K/Akt and the Raf/MAPK pathways, which promote cell proliferation and inhibit apoptosis (Pollak et al., 2004).

DM and obesity are associated with other hormonal alterations that may promote neoplasia as well. Adiponectin is a hormone that has been shown to limit angiogenesis, promote apoptosis, and decrease inflammation. DM is associated with low circulating levels of adiponectin, an effect that may promote carcinogenesis (Bao et al., 2011). Leptin is a mitogenic hormone which is increased in obesity. It promotes angiogenesis, inhibits apoptosis, and activates the PI3K/Akt and STAT pathways, which promote cell growth and survival (Bao et al., 2011).

### Pancreatic cancer-associated diabetes mellitus

Several studies have suggested that DM is not just a risk factor, but also a consequence of pancreatic cancer. One study looked at the temporal association between DM and pancreatic cancer and found a marked increase in cases of DM starting at 36 months prior to diagnosis, with cases continuing to increase up to cancer diagnosis, suggesting that the cancer itself was likely the etiologic factor (Chari et al., 2008). A meta-analysis of 35 cohort studies showed DM was associated with a 94% increased risk of pancreatic cancer. Interestingly, risk decreased with duration of diabetes (5.38 for <1 year, 1.95 for 1–4 years, 1.49 for 5–9 years, 1.47 for ≥10 years) providing support that much of diabetes in pancreatic cancer patients is caused by the cancer itself (Ben et al., 2011).

Pancreatic cancer-induced DM is thought to be a paraneoplastic phenomenon involving release of products from the tumor rather than a result of destruction of the pancreas due to malignant infiltration (Pannala et al., 2008; Aggarwal et al., 2012). This hypothesis is supported by a study which showed that the prevalence of DM in patients with pancreatic cancer was not related to tumor stage or location, as would be expected if the DM were a result of tumor infiltration (Pannala et al., 2008). Furthermore, several studies have shown resolution of DM after tumor resection in individuals with pancreatic cancer (Permert et al., 1993a; Fogar et al., 1994; Pannala et al., 2008; White et al., 2011). A study by Aggarwal et al. (2012) showed that the hormone adrenomedullin is upregulated at the mRNA and protein level in pancreata from patients with pancreatic cancer. *In vitro* studies indicate that adrenomedullin impairs glucose-stimulated insulin secretion in β-cells, and adrenomedullin overexpression in mouse pancreatic cancer tissues is associated with increased glucose intolerance suggesting that adrenomedullin is an important mediator of cancer-induced DM (Aggarwal et al., 2012). Other potential mediators found upregulated in pancreatic cancer include S-100A8 N-terminal peptide, which was shown to alter glucose catabolism *in vitro* (Basso et al., 2006), and islet amyloid polypeptide which causes insulin resistance (Permert et al., 1994). One review suggested that pancreatic cancer-associated insulin resistance may be mediated by release of cytokines, adipokines, and non-esterified fatty acids from adipose tissue inflammation (Sah et al., 2013).

## Standards of therapy

Surgical resection is the only cure for pancreatic adenocarcinoma although 80% of patients have unresectable disease at the time of presentation (Campen et al., 2011). Pancreaticoduodenectomy and distal pancreatectomy with or without splenectomy are performed in patients with tumors in the head and body/tail of the pancreas, respectively (De La Cruz et al., 2014). Given that median survival is poor even in patients that undergo surgical resection, most patients are offered adjuvant chemotherapy with gemcitabine or fluorouracil ± chemoradiation (De La Cruz et al., 2014).

Patients with locally advanced, unresectable, or borderline resectable disease may receive neoadjuvant chemotherapy ± radiotherapy in an attempt to downstage the disease (Seufferlein et al., 2012). For metastatic disease, treatment options address palliation and improved survival. Gemcitabine has been used to treat metastatic pancreatic adenocarcinoma since 1997 when it produced a one year survival benefit compared to 5-FU and remains the chemotherapy of choice in patients with poor functional status and advanced disease (Burris et al., 1997; Ghosn et al., 2014). In 2011, FOLFIRINOX (fluorouracil, leucovorin, irinotecan, and oxaliplatin) produced a significant survival benefit compared to patients treated with gemcitabine monotherapy (Conroy et al., 2011). Gemcitabine plus nab-paclitaxel produces less adverse effects than FOLFIRINOX and offers the second best overall survival in those with good functional status (Ghosn et al., 2014). Gemcitabine plus erlotinib shows improved survival vs. monotherapy, but survival benefit remains minimal (Ghosn et al., 2014). Despite the fact that chemotherapy confers some survival benefit, this benefit is modest and exploration of new therapies is essential.

## Metformin and pancreatic cancer

### Metformin reduces risk for pancreatic cancer

Metformin is one of the most widely prescribed oral agents for the treatment of DM2. Evans et al. (2005) were among the first to suggest metformin as an anti-cancer therapy. However, as early as 2001, one study demonstrated that metformin prevented pancreatic cancer development in hamsters treated with a pancreatic carcinogen (Schneider et al., 2001). A study in 2009 with 62,809 patients compared cancer risk in patients on different kinds of diabetic therapies and found that insulin therapy increased the risk of pancreatic cancer compared to metformin therapy (Currie et al., 2009). Furthermore, compared to patients that were on insulin alone, patients with metformin added to insulin had a significantly reduced risk of developing solid tumors (Currie et al., 2009). A recent meta-analysis showed that patients with diabetes who were taking metformin had a significantly reduced risk of pancreatic cancer (Wang et al., 2014). Consistent with this finding, an observational study of 302 diabetic patients with pancreatic cancer found that metformin users showed significantly increased survival times as compared to non-users (15.2 months vs. 11.1 months, Sadeghi et al., 2012).

### Mechanisms of action

Although there is much to be learned about metformin's mechanism of action in cancer (Figure 1), most studies suggest that metformin acts primarily through its effect on AMP-activated protein kinase (AMPK, Kalender et al., 2010; Ben Sahra et al., 2011). Metformin inhibits complex I of the electron transport chain (El-Mir et al., 2000), which decreases adenosine triphosphate (ATP) production and leads to AMPK activation. AMPK activation leads to disruption of insulin/IGF-1 signaling through inhibition of mammalian target of rapamycin (mTOR, Rozengurt et al., 2010). Inhibition of mTOR signaling, in turn, results in decreased protein synthesis and cell growth, which are important for cancer survival (Figure 1). Metformin can also inhibit mTOR signaling through activation of AMPK independent pathways including Rag GTPase (Kalender et al., 2010) and REDD1 (Ben Sahra et al., 2011). AMPK-induced activation of tumor suppressor 53 (p53) and subsequent cell cycle arrest represents another potential mechanism of action of metformin in pancreatic cancer models (Jalving et al., 2010). Activation of AMPK in the liver, muscle, adipose tissue, and pancreas also results in reduced levels of insulin and IGF-1 (Jalving et al., 2010).

Indeed, mTOR and mitochondrial energy signaling pathways have increasingly been the focus of recent investigations on the antitumor properties of metformin in pancreatic cancer. Early studies demonstrated that metformin induced inhibition of mTORC1 activity and growth of human pancreatic cancer xenografts (Kisfalvi et al., 2009). Concentrations of metformin approximate to plasma levels found in patients with DM2 taking the drug (0.05 mM or 0.1 mM) similarly inhibited mTORC1 activity in a dose-dependent manner *in vitro* (Sinnett-Smith et al., 2012). Interestingly, metformin-induced mTORC1 inhibition was significantly enhanced in pancreatic cancer cells cultured in physiologic glucose concentrations (5 mM) compared to supra-physiologic levels (25 mM) highlighting the concept that cancer cells, at lower ambient glucose concentrations, rely more on mitochondrial oxidative phosphorylation for ATP generation and are therefore more sensitized to mitochondrial respiration inhibitors (Sinnett-Smith et al., 2012; Rozengurt, 2014). Furthermore, synergistic enhancements in ATP depletion and pancreatic cancer cell growth suppression were demonstrated when metformin was added to an inhibitor of glycolysis, 2-deoxyglucose (2-DG), *in vitro* (Cheng et al., 2014).

Recent studies have shown that pancreatic cancer cell growth (*in vitro* and *in vivo*) is also dependent on glutamine metabolism reprogrammed by oncogenic *Kras* via a unique pathway involving aspartate transaminase (GOT1) that leads to a maintenance of essential cellular redox states in the mitochondria (Son et al., 2013). Moreover, pancreatic cancer cells responsible for relapse that survive oncogene ablation have increased expression of genes involving mitochondrial function and reliance on glycolysis and mitochondrial respiration for energy metabolism in *Kras* mouse models (Viale et al., 2014). These latest insights offer exciting future therapeutic strategies in pancreatic cancer by targeting *Kras* signaling in combination with using mitochondrial respiration inhibitors such as metformin. Further identification of novel therapeutic targets will rely, as they have before, on the development of tools critical to our understanding of pancreatic cancer such as genetically engineered mouse models (GEMMs) like the *Kras*^*G*12*D*^ and embryonic stem cell (ESC)-based mouse models (Kirk, 2012; Saborowski et al., 2014).

Rapamycin and active-site mTOR inhibitors have been shown to increase Akt phosphorylation and ERK activation in pancreatic cancer cells *in vitro*, respectively, and highlight the feedback mechanisms that potentially counterbalance the antitumor effects of mTOR inhibitors (Soares et al., 2013). Metformin's antitumor effects, however, occur without stimulating Akt and ERK activation, and therefore, metformin in combination with mTOR inhibitors represents a future direction to improve clinical efficacy in pancreatic cancer (Soares et al., 2013). Indeed, metformin with rapamycin is now under ongoing clinical investigation (phase I and II) in the treatment of advanced pancreatic cancer (NCT02048384). Of note, PTEN deficiencies in *Kras*^*G*12*D*^ mice models have been shown to promote NF-κ B and associated cytokine activation and development of metastatic pancreatic cancer (Ying et al., 2011). Treatment with rapamycin conferred a significant survival advantage in *Kras* mice models deficient of PTEN compared to those lacking PTEN deficiencies *in vivo* (Morran et al., 2014). These studies intriguingly identify particular subsets of pancreatic cancer, those with *Kras* mutations and PTEN deficiencies, that may be more responsive to treatment with mTOR inhibitors and/or inhibitors of MAPK/ERK, PI3K, and NF-κ B (mediators of converging signaling pathways).

Other studies have shown that metformin treatment leads to downregulation of members of the specificity protein (Sp) transcription factor family and target genes involved in tumorigenesis including Bcl-2, survivin, cyclin D1, vascular endothelial growth factor, and fatty acid synthase (FAS, Nair et al., 2013, 2014). In particular, decrease in cyclin D1 induced cell cycle arrest in prostate cancer cells (Ben Sahra et al., 2008). Metformin may also inhibit FAS in the context of available cholesterol and glucose-derived acetyl-CoA in pancreatic cancer cells (Cantoria et al., 2014). In addition, *in vitro* studies showed that metformin alters profiles of microRNAs that regulate apoptosis, inhibit proliferation and invasion, and are linked to reduced expression of the oncogene HMGA1 (Li et al., 2012). Metformin can also affect proliferation of cancer stem cells, and this effect may contribute to its ability to limit tumor growth (Gou et al., 2013). Other potential anti-tumorigenic effects of metformin include the ability to reduce endogenous reactive oxygen species (ROS) and associated DNA damage (Algire et al., 2012), reduce Sonic hedgehog (Shh) expression (Nakamura et al., 2014), and induce anti-inflammatory responses (Cufí et al., 2010; Hirsch et al., 2013; Zhao et al., 2014).

Metformin has demonstrated antitumor activity against pancreatic cancer in numerous preclinical studies (Table 1). A recent study highlighted the ability of metformin to prevent progression of pancreatic intraepithelial neoplasia (PanIN) lesions to pancreatic cancer in transgenic mice as well (Mohammed et al., 2013). There are several clinical trials (phase I and II, https://clinicaltrials.gov) involving metformin in the treatment of pancreatic cancer. The majority involve metformin in combination therapies, given that metformin is unlikely to produce a desired efficacy to serve as monotherapy in pancreatic cancer. However, its attractiveness as part of combinatorial therapy lies, in part, in its inexpensiveness (as low as $4 per month) and well-tolerated toxicity profile (common toxicities being gastrointestinal). As discussed above, metformin offers synergistic activity across several but converging signaling pathways important to tumorigenesis. Addition of metformin may also reduce effective doses of other chemotherapeutic agents needed to treat a variety of cancers (Iliopoulos et al., 2011).

**Table 1:** **The preclinical development of metformin in pancreatic cancer**.

**Study**	**Source in which metformin demonstrated antitumor activity**
Cheng et al., 2014	MiaPaCa-2 and Capan-2 cells (*in vitro*, ± glycolytic inhibitor 2-DG)
Das et al., 2014	Panc-1 and AsPC-1 cells (*in vitro*)
Fasih et al., 2014	Panc-1 and MiaPaCa-2 cells (*in vitro*, ± ionizing radiation ± gemcitabine)
Nair et al., 2014	Panc-28, L3.6pL, and Panc-1 cells (*in vitro*)
Snima et al., 2014	MiaPaCa-2 cells (*in vitro*, in metformin-containing *O*-carboxymethyl chitosan nanoparticles)
Yue et al., 2014	BxPC-3 and Panc-1 cells (*in vitro*, + aspirin)
Gou et al., 2013	AsPC-1 and SW1990 cells (*in vitro*) and AsPC-1 and SW1990 tumor xenografts in nude mice (*in vivo*)
Karnevi et al., 2013	BxPC-3, Panc-1, and MiaPaCa-2 cells (*in vitro*)
Kisfalvi et al., 2013	Panc-1 and MiaPaCa-2 tumor xenografts in nude mice (*in vivo*)
Lonardo et al., 2013	Primary human pancreatic ductal adenocarcinoma (PDAC) cells and spheres (*in vitro*, ± gemcitabine) and tumor xenografts in nude mice (*in vivo*, ± gemcitabine and smoothened inhibitor SIBI-C1)
Nair et al., 2013	Panc-28, Panc-1, and L3.6pL cells (*in vitro*) and L3.6pL tumor xenografts in nude mice (*in vivo*)
Soares et al., 2013	Panc-1 cells (*in vitro*)
Yan et al., 2013	MiaPaCa and KP cells (*in vitro*)
Bao et al., 2012	Gemcitabine-sensitive and gemcitabine-resistant AsPC-1 and MiaPaCa-2 cells (*in vitro*) and MiaPaCa-2 tumor xenografts in mice (*in vivo*)
Li et al., 2012	Sw1990 tumor xenografts in nude mice (*in vivo*)
Sinnett-Smith et al., 2012	Panc-1 and MiaPaCa-2 cells (*in vitro*)
Snima et al., 2012	MiaPaCa-2 cells (*in vitro*, in metformin-containing *O*-carboxymethyl chitosan nanoparticles)
Kisfalvi et al., 2009	Panc-1 and MiaPaCa-2 tumor xenografts in nude mice (*in vivo*)
Wang et al., 2008	SW1990, AsPC-1, BxPc-3, and Panc-1 cells (*in vitro*)

Undoubtedly, there remains a need for further understanding of: (1) anticancer mechanisms of metformin, particularly those involving, but not limited to, mTOR signaling (upstream and downstream) and mitochondrial energy metabolism, (2) pharmacokinetic and pharmacodynamic properties of metformin, and (3) relationships between risk factors such as DM and development and progression of pancreatic cancer to identify further molecular targets and advance potential therapies. For now, the future remains bright for metformin as the scientific community eagerly awaits the results of its continued development as a treatment for pancreatic cancer.

### Conflict of interest statement

The Guest Associate Editor Guido Eibl declares that, despite being affiliated to the same institution as author Stephen J. Pandol and having collaborated with him, the review process was handled objectively and no conflict of interest exists. The authors declare that the research was conducted in the absence of any commercial or financial relationships that could be construed as a potential conflict of interest.
